# Nota editorial en tiempos de la pandemia por SARS-CoV-2

**Published:** 2020-11-11

**Authors:** Luis Alberto Gómez

**Affiliations:** 1 Bogotá, D.C., Colombia Editor, revista Biomédica; investigador, Grupo de Fisiología Molecular, Instituto Nacional de Salud; profesor catedrático, Departamento de Ciencias Fisiológicas, Facultad de Medicina, Universidad Nacional de Colombia; miembro correspondiente, Academia Colombiana de Ciencias Exactas Físicas y Naturales Universidad Nacional de Colombia Facultad de Medicina Universidad Nacional de Colombia BogotáD.C. Colombia

En la revista *Biomédica* se le está dando prioridad a las contribuciones originales sobre el virus SARS-CoV-2 y la infección de COVID-19, dada la urgencia y el impacto de la pandemia en Colombia y en todo el mundo. Desde que empezó la emergencia hemos continuado nuestra labor manteniendo los altos estándares de publicación de la revista.

Agradezco los manuscritos recibidos hasta el momento y los que esperamos recibir, así como el trabajo de los editores, los evaluadores y el equipo editorial, y espero que nuestros autores hagan gala de tolerancia en aras de la crítica científica constructiva. Los editores de la revista supervisaremos el proceso de revisión y evaluación para garantizar que las solicitudes de trabajo adicional para la evaluación de los manuscritos estén dentro de lo razonable. Lo hacemos juiciosa y cuidadosamente, ponderando su valor a partir del estado en que son recibidos, la probabilidad de que los autores completen las modificaciones solicitadas en un plazo razonable y la necesidad y el valor científico agregado de los experimentos, los datos y las sustentaciones.

Dependemos totalmente de la comunidad de evaluadores y editores para mantener los estándares de la revista y para ayudar a los autores a mejorar sus manuscritos. Reconozco que muchos evaluadores, editores e integrantes del equipo editorial pueden ver su carga de trabajo aumentada durante este tiempo de emergencia, ya sea en relación con su trabajo o con sus obligaciones investigativas, docentes, administrativas o familiares.

La investigación biomédica sobre COVID-19 es prioritaria y por ello la mayoría de las editoriales y revistas científicas del mundo están permitiendo el acceso abierto de las publicaciones relacionadas con la pandemia, reconociendo la necesidad de compartir información científica sobre el coronavirus causante de la enfermedad, el SARS-CoV-2. Al final de esta nota se recomiendan algunas de las principales fuentes de información de acceso abierto y gratuito ^1^^-^^9^ que pueden facilitar la apropiación social del conocimiento actual en el tema.

Asimismo, soy consciente de que esta pandemia puede causar interrupciones en el trabajo de nuestra comunidad de científicos. Analizo esta situación en mi calidad de editor, y una de mis principales preocupaciones es garantizar que la comunicación de los resultados y avances científicos en el campo de la biomedicina continúe de la mejor manera posible y se mantenga durante esta emergencia.

También quiero informarles a nuestros colaboradores, evaluadores y lectores algunas opciones disponibles con las que cuenta *Biomédica.* Llamo la atención de quienes contemplan la presentación de trabajos nuevos en torno a los temas de la revista, en particular sobre SARS-CoV-2 y COVID-19, en el sentido de que si sus contribuciones son científicamente sólidas y novedosas, estamos preparados para acelerar su revisión y publicarlas en el menor tiempo posible una vez sean aprobadas para publicación anticipada ^10^.

Es posible que algunos investigadores tengan resultados novedosos y de gran mérito que, sin embargo, requerirían un trabajo adicional considerable para llegar al nivel de un artículo completo. Si el cronograma de finalización de la investigación es muy incierto, o está proyectado para una fecha lejana en el futuro, pero es imperativo comunicar los resultados parciales de manera oportuna, pueden considerar el envío de su estudio a la revista en la categoría de comunicación breve.

Por último, quiero invitarlos a hacer uso de las herramientas virtuales y digitales dispuestas para cumplir con todos los pasos involucrados en el proceso editorial en la plataforma de la revista *Biomédica*^10^^,^^11^, especialmente en estos tiempos de la pandemia por el virus SARS-CoV-2.
